# Atypical Hand, Foot, and Mouth Disease in an Immunocompetent Adult

**DOI:** 10.7759/cureus.109835

**Published:** 2026-05-28

**Authors:** Emma B Carpenter, Caroline Lunny, Emma Leone, Alexander M Hammond, Kiran Motaparthi, Charlotte Chaiklin

**Affiliations:** 1 Department of Medicine, University of Florida College of Medicine, Gainesville, USA; 2 Department of Neurology, University of Florida, Gainesville, USA; 3 Department of Dermatology, University of Florida, Gainesville, USA; 4 Department of Medicine, University of Florida, Gainesville, USA

**Keywords:** and mouth disease in adults, coxsackievirus, dermatology, foot, hand, infectious diseases, viral exanthem

## Abstract

Hand, foot, and mouth disease (HFMD) is a common viral illness of childhood but is increasingly recognized in adults with often atypical and severe presentations. We report a case of HFMD in an immunocompetent man in his sixties who presented with a generalized, tender vesiculopapular eruption involving the groin, trunk, extremities, palms, and soles. An extensive infectious evaluation was initially pursued, and empiric broad-spectrum antimicrobials were administered. Dermatology consultation led to the diagnosis of HFMD, and antibiotics were discontinued with subsequent clinical improvement. This case highlights the importance of considering HFMD in the differential diagnosis of vesiculopapular eruptions in adults, particularly when the palms and soles are involved, to avoid unnecessary antimicrobial exposure and invasive testing.

## Introduction

Hand, foot, and mouth disease (HFMD) is a highly contagious viral illness that most commonly affects children under 10 years of age [1]. The disease classically presents with a low-grade fever and painful oral ulcerations followed by a polymorphous exanthem consisting of erythematous macules, papules, and tense vesicles often involving the palms and soles [1]. HFMD is traditionally caused by coxsackievirus A16 and enterovirus A71, though multiple enterovirus serotypes can cause the disease, including coxsackievirus A6 and coxsackievirus B serotypes [1-5].

While HFMD in immunocompetent adults is rare, cases have increased in recent years, often linked to household transmission from children and to emerging viral serotypes [2-4,6-9]. Adult cases may present with more extensive cutaneous involvement, severe pain, and rare complications such as onychomadesis and cardiomyopathy [6-10]. Epidemiological data suggest that while HFMD is not a prevalent health problem in adults, its incidence is rising and may pose a potential public health threat, especially in regions experiencing outbreaks or viral evolution [6-7]. We present a case of atypical HFMD in an immunocompetent adult with widespread vesiculopapular eruption and systemic symptoms, underscoring the diagnostic challenges and importance of recognizing this entity in adults.

## Case presentation

A man in his sixties with a history of glaucoma presented to the emergency department during the summer with five days of a progressively worsening rash and sore throat. The eruption initially appeared in the groin as tender, skin-colored papules without surrounding erythema. Over several days, the eruption evolved into erythematous macules, papules, and vesicles, becoming more generalized, involving the groin, buttocks, trunk, extremities, palms, and soles.

Associated symptoms included odynophagia, chills, decreased appetite, constipation, bilateral wrist and elbow pain, and a transient sensation of throat swelling that resolved spontaneously. The patient reported recent exposure to children at a social gathering about one week prior to symptom onset. He denied recent tick bites or new sexual partners. His wife was asymptomatic. Childhood illnesses included possible measles or varicella infection, and he reported remote recurrent herpes labialis.

**Figure 1:**
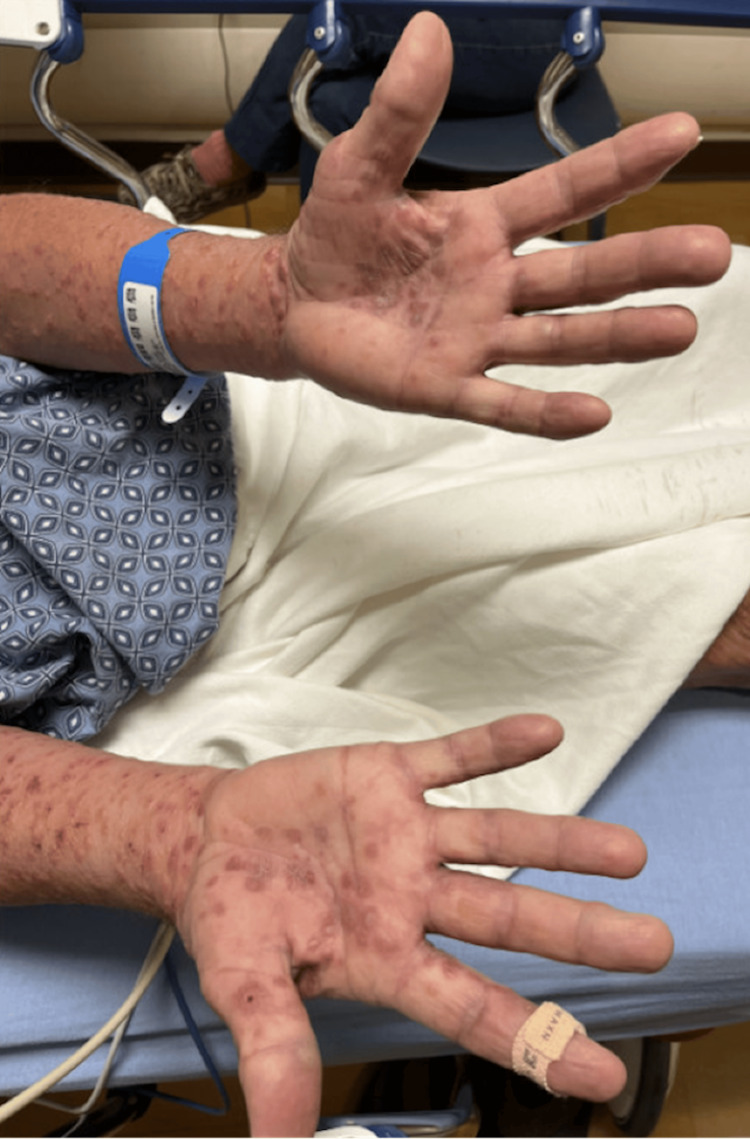
Erythematous, red-brown football-shaped papules and vesicles - some with hemorrhagic crust - on palmar surfaces of bilateral hands at presentation.

**Figure 2:**
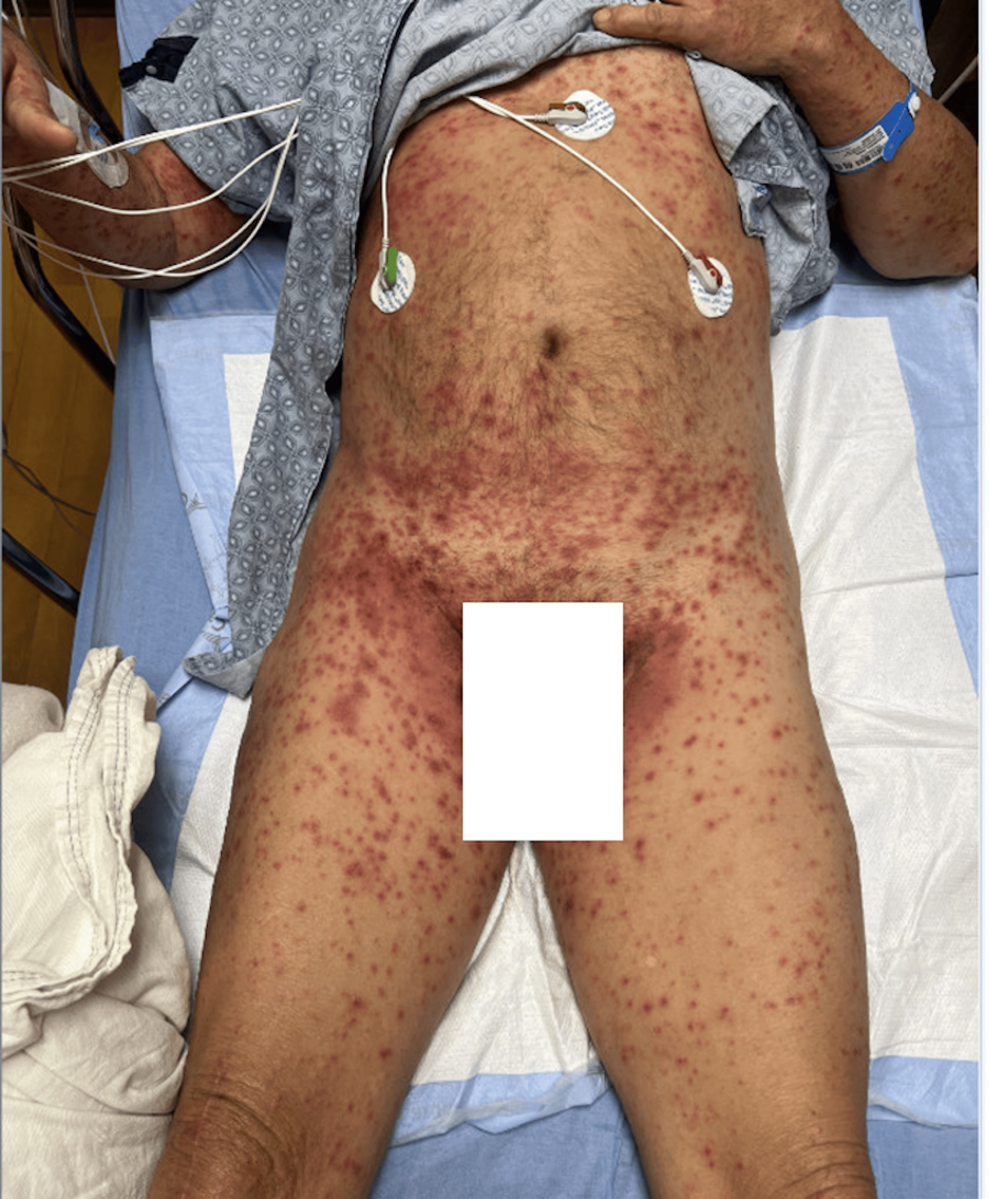
Widespread, polymorphic erythematous, red-brown macules, papules, vesicles - some with hemorrhagic crust - on the trunk, groin, and lower extremities at presentation.

On presentation, he was afebrile with mild tachycardia, normotension, and normal oxygen saturation on room air. Physical examination revealed generalized red-brown, football-shaped macules, papules, and hemorrhagic tense vesicles - some with secondary crust - involving the face, trunk, groin, buttocks, palms, and soles (Figures 1, 2). Lip crusting and tender cervical lymphadenopathy were noted. The left conjunctiva was mildly injected. The oropharynx was erythematous without visible mucosal lesions. Serous drainage was present from the left ear, with normal-appearing tympanic membranes bilaterally.

Laboratory evaluation demonstrated leukocytosis, elevated hemoglobin, hyponatremia, and elevated inflammatory markers, including C-reactive protein and erythrocyte sedimentation rate (Table 1). Given systemic symptoms and inflammatory markers, the patient was admitted and empirically treated with vancomycin, cefepime, and doxycycline due to concerns for infection.

**Table 1:** Laboratory findings on admission

Parameter	Patient Value	Reference Range
White blood cell count	21.2 × 10⁹/L	4.0–10.0 × 10⁹/L
Hemoglobin	18.3 g/dL	13.0–16.5 g/dL
Sodium	129 mmol/L	135–145 mmol/L
Creatinine	1.2 mg/dL	0.51–1.18 mg/dL
C-reactive protein	108 mg/L	0–5 mg/L
Erythrocyte sedimentation rate	23 mm/hour	0–10 mm/hr

**Figure 3:**
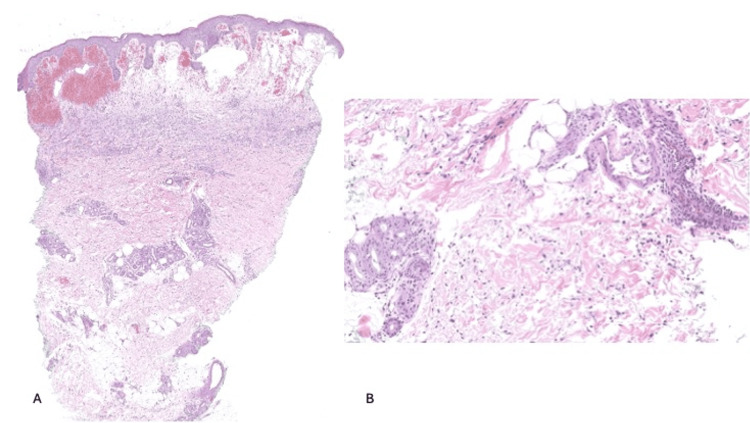
Massive superficial dermal edema, along with a perivascular, periadnexal, and interstitial mixed infiltrate (A: H&E, 20x magnification & B: H&E, 200x magnification).

**Figure 4:**
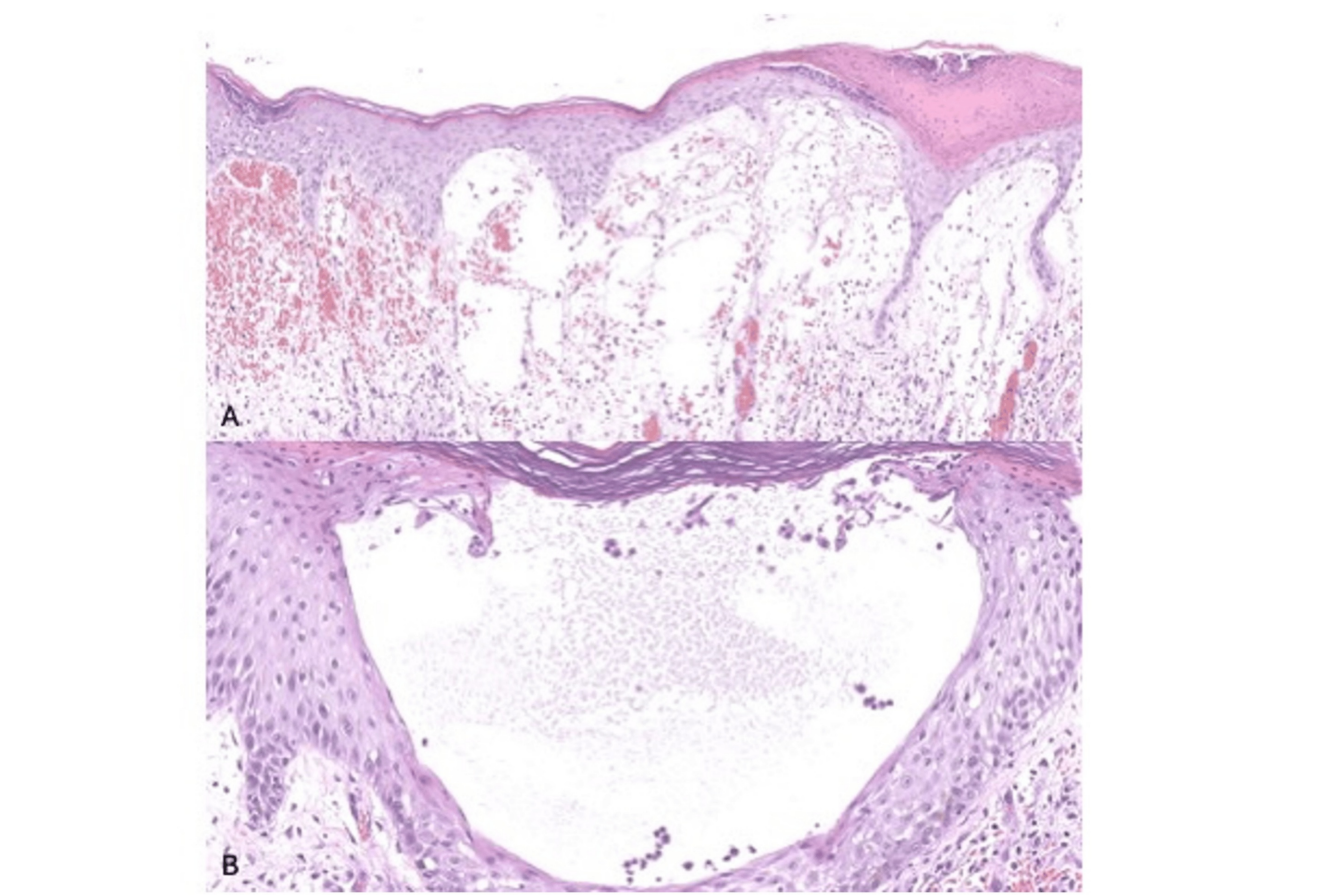
Crust and necrosis limited to the upper epidermis with marked spongiosis including vesiculation with neutrophils (A: H&E, 40x magnification & B: H&E, 200x magnification).

An extensive infectious evaluation was pursued, including testing for human immunodeficiency virus, syphilis, Lyme disease, rheumatic disease secondary to group A Streptococcus, rickettsial infections, Mpox, and fungal and zoonotic pathogens including Histoplasma, Brucella, and Toxoplasma; all results were negative. Varicella-zoster DNA polymerase chain reaction (PCR) and SARS-CoV-2 PCR were both negative. Following consultation, the dermatology inpatient service made the clinical diagnosis of HFMD based on the patient’s preceding enanthem, lesion morphology, and distribution, including involvement of the palms and soles. To minimize additional testing for other infections and to provide rapid diagnostic confirmation in the inpatient setting, punch biopsies of skin were obtained. Histopathology demonstrated necrotic keratinocytes limited to the upper third of the epidermis, intracytoplasmic and intercellular edema, and an absence of viral cytopathic change - nonspecific but consistent and typical histopathologic findings of HFMD (Figures 3, 4). Serologic testing demonstrated elevated total antibody titers to coxsackievirus B3, B4, and B5; however, because the assay did not distinguish between IgG and IgM antibodies, interpretation in the acute clinical setting was limited. Testing for coxsackievirus A6 was not performed due to lack of availability. After a multidisciplinary discussion with dermatology and infectious disease, the patient was ultimately diagnosed with HFMD.

**Figure 5:**
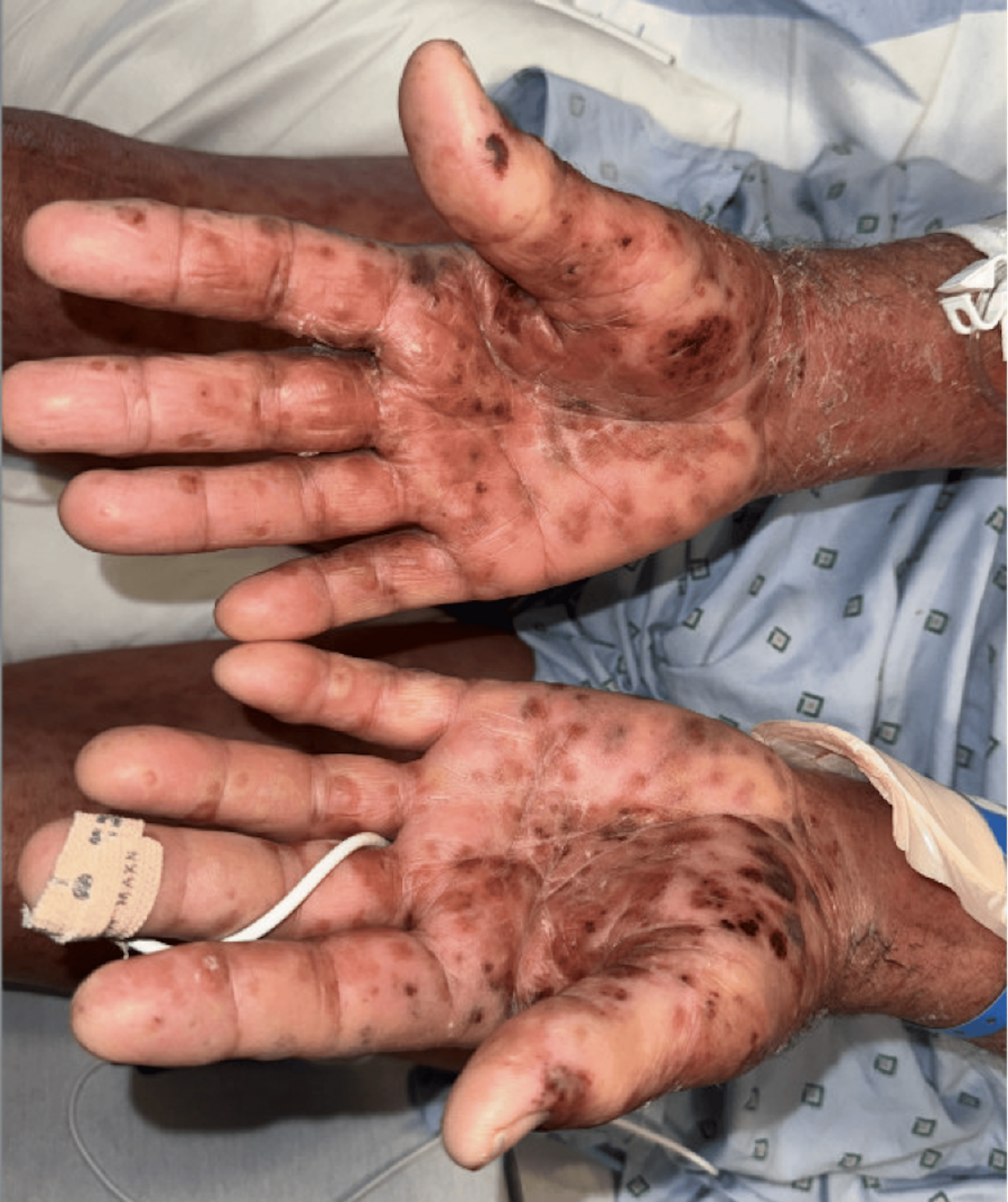
Scattered polymorphous red-brown or hemorrhagic tense vesicles on palmar surfaces of bilateral hands with proximal desquamation at the wrists on hospital day 4.

**Figure 6:**
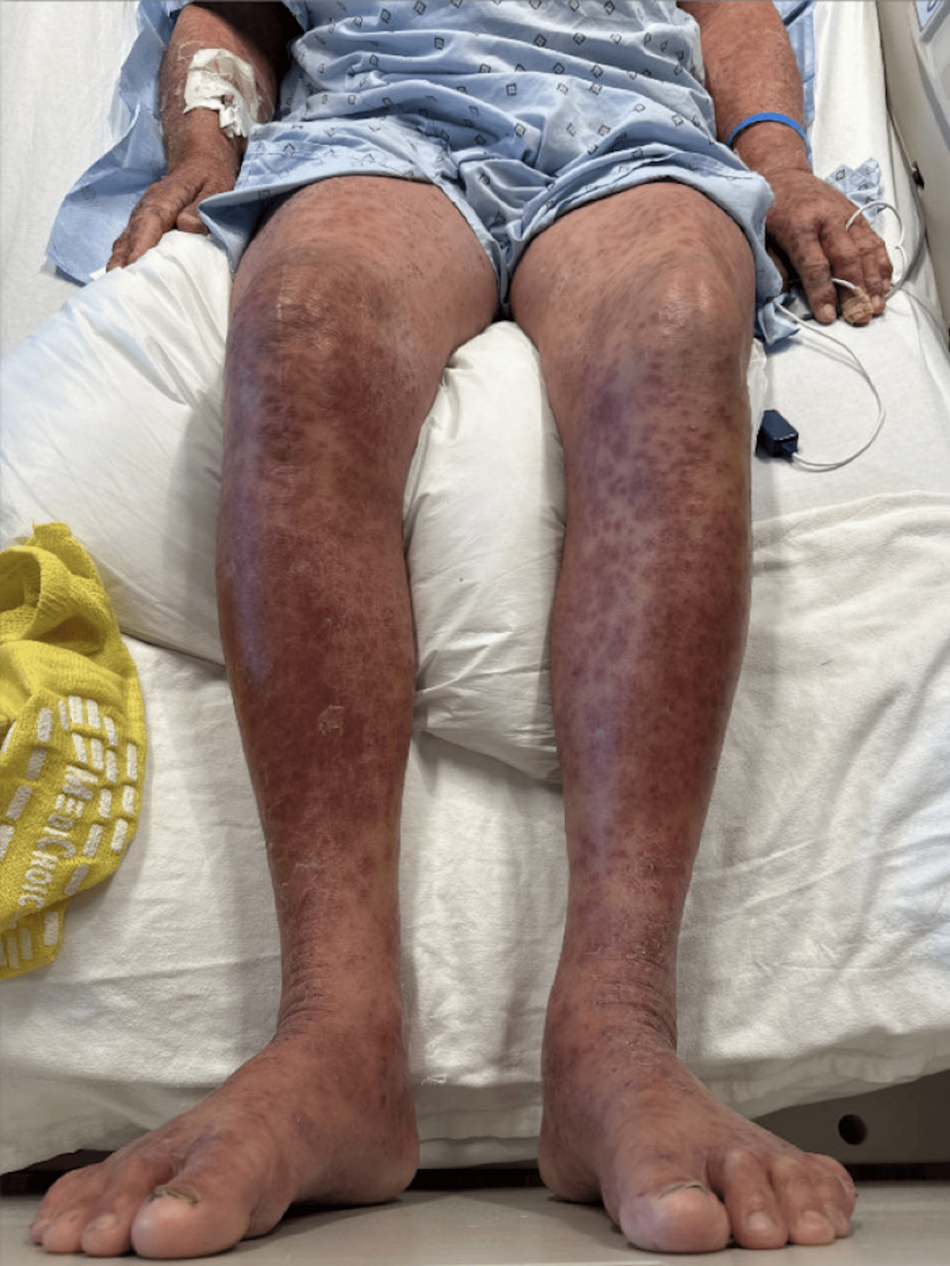
Coalescing scattered polymorphous red-brown or hemorrhagic tense vesicles and macules of the bilateral lower extremities on hospital day 4.

**Figure 7:**
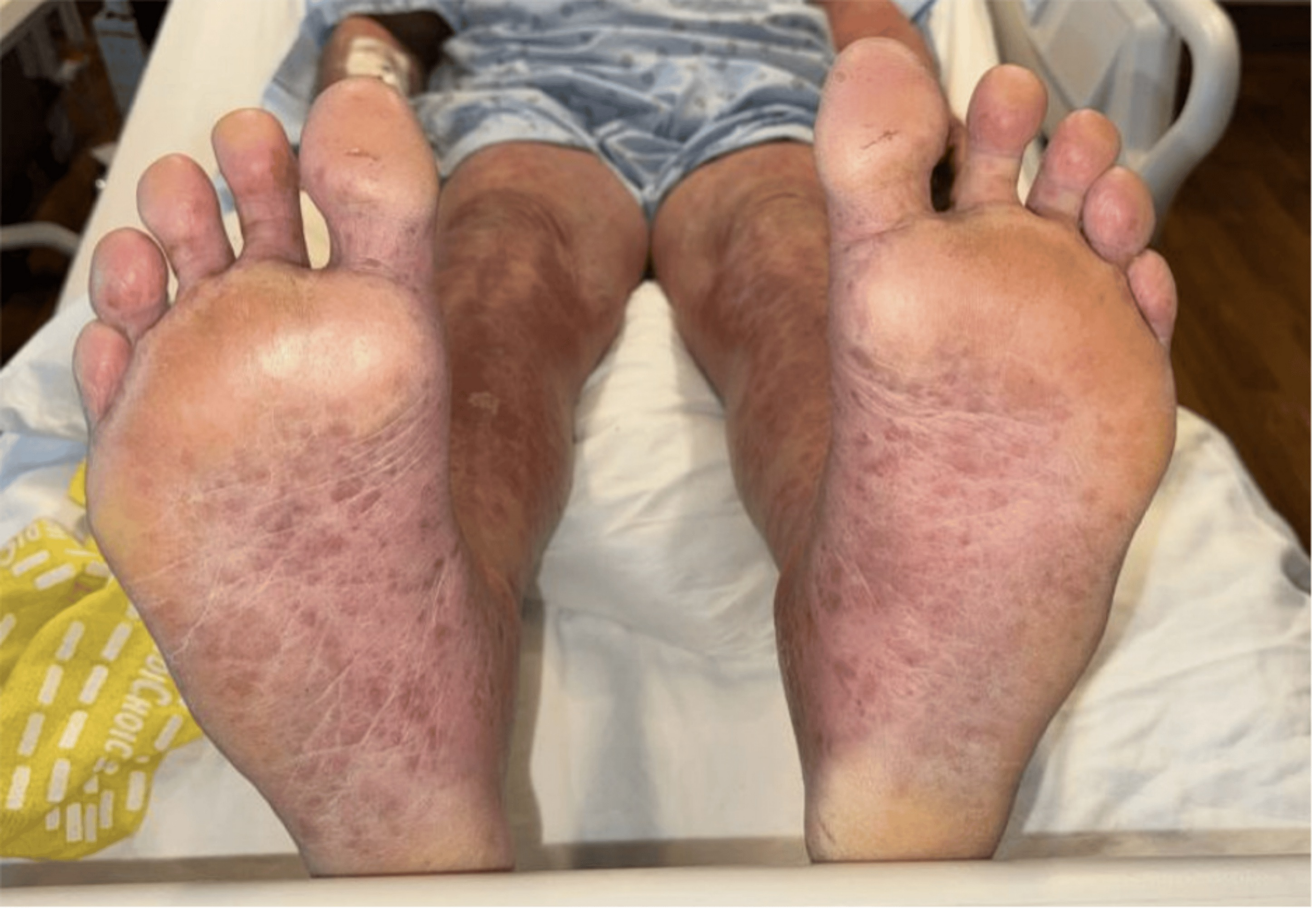
Scattered polymorphous red-brown or hemorrhagic tense vesicles and macules on plantar surfaces and toes of bilateral feet with desquamation on hospital day 4.

**Figure 8:**
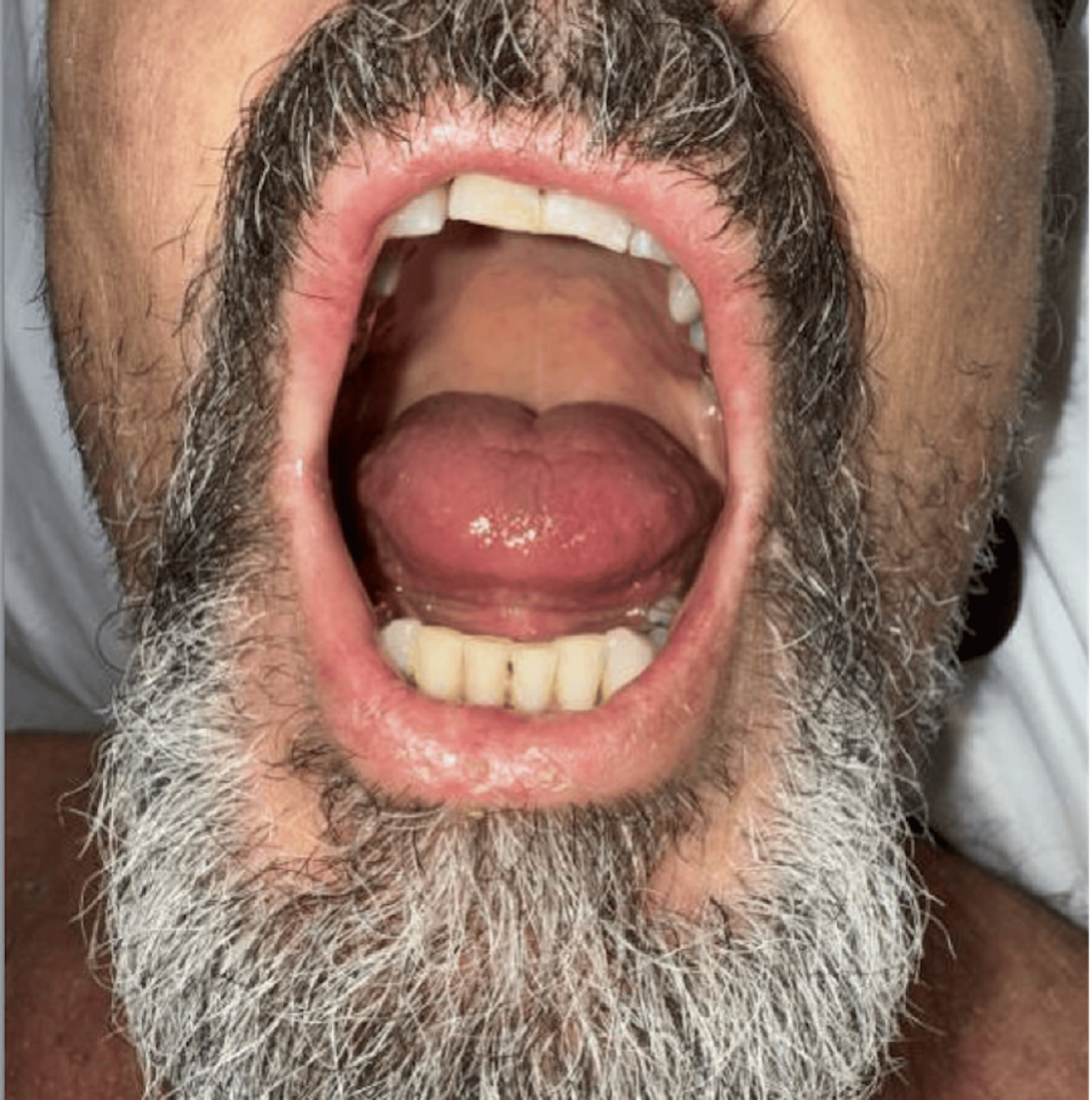
Oral mucosa and posterior oropharynx without visible lesions on hospital day 4.

Antimicrobial therapy was discontinued, and the patient was managed with supportive care, including intravenous fluids, acetaminophen, and gabapentin for pain control. His hyponatremia, acute kidney injury, and hemoconcentration were attributed to poor oral intake in the setting of odynophagia and resolved with hydration and improved nutrition. Cutaneous lesions continued to evolve but gradually improved, with resolution of systemic symptoms without any further antimicrobial therapy (Figures 5-8). The patient was discharged home in stable condition, and the lesions subsequently crusted and resolved completely over the next few weeks.

## Discussion

HFMD is classically a self-limited viral exanthem of childhood characterized by vesicular eruptions on the hands, feet, and oral mucosa, with typical resolution in 7 to 10 days [1]. Adult presentations, though uncommon and atypical, are increasing [2,3,7-9]. Surveillance data from the Centers for Disease Control and Prevention (CDC) demonstrated that in the 2011-2012 outbreak of severe HFMD, approximately 25% of reported cases occurred in adults with close contact to infected children [3]. Subsequent outbreak reports continue to document a rising prevalence of adult HFMD in association with highly virulent coxsackievirus serotypes [2,3,8,9].

Clinically, adult HFMD presents as widespread vesiculobullous eruptions involving atypical sites such as the face, scalp, and trunk and may be accompanied by fever, arthralgia, and severe pain necessitating hospitalization [3,8]. The primary mode of adult acquisition is direct physical contact with infected individuals, most commonly children. Other routes of exposure include saliva, respiratory secretions, vesicular fluid, or stool [1]. Patients are contagious until all cutaneous lesions are crusted over. Indirect exposure via contaminated surfaces and fomites is also reported [1,4]. Close contact with children is the strongest risk factor for adult infection [6,9]. Suboptimal personal hygiene practices, particularly inadequate handwashing, further increase susceptibility [1].

Management of adult HFMD is supportive and includes adequate hydration, analgesia, and infection-control measures, as no antiviral therapy is currently available [1]. Recognition of atypical HFMD in adults is critical to prevent diagnostic error, avoid unnecessary or prolonged antimicrobial therapy, and reinforce effective hygiene practices to limit transmission.

## Conclusions

This case report highlights an atypical presentation of HFMD in an immunocompetent adult with extensive vesiculopapular involvement and systemic symptoms that initially prompted consideration of alternative infections. The patient's widespread cutaneous eruption, involvement of the palms and soles, and systemic manifestations illustrate the increasingly recognized spectrum of adult HFMD presentations that are often associated with emerging viral serotypes.

Recognition of these atypical manifestations is important for clinicians evaluating diffuse vesicular or polymorphic rashes in adults, especially when other viral exanthems such as herpes simplex virus (HSV), varicella zoster virus (VZV), or Mpox are being considered. In this context, bedside Tzanck smear is a helpful, inexpensive, and widely available test that can be used to rapidly exclude HSV, VZV, and Mpox when negative for cytopathic change. Histopathologic evaluation can also provide timely supportive evidence for diagnosis when features are consistent with HFMD. Although serologic testing is noninvasive, its utility in the acute setting is limited by slow turnaround times and the inability of total immunoglobulin testing alone to reliably distinguish prior infection from recent infection. To avoid unnecessary diagnostic evaluation, serologic testing should not be used when rapid confirmation of HFMD is desired. Increased awareness of adult HFMD may help reduce diagnostic delay, avoid unnecessary diagnostic testing and treatment, and allow for appropriate supportive management. Continued reporting of atypical adult presentations may further clarify the evolving epidemiology and clinical spectrum of this traditionally pediatric illness.
